# Substance P induces sympathetic immune response in the contralateral eye after the first eye cataract surgery in type 2 diabetic patients

**DOI:** 10.1186/s12886-020-01598-4

**Published:** 2020-08-18

**Authors:** Xianhui Gong, Yueping Ren, Xiuxiu Fang, Junyong Cai, E. Song

**Affiliations:** 1 The Second Affiliated Hospital of Soochow University, Suzhou, Jiangsu China; 2 Lixiang Eye Hospital of Soochow University, Suzhou, Jiangsu China; 3 The Affiliated Eye Hospital of Wenzhou Medical University, Wenzhou, Zhejiang China

**Keywords:** Substance P, MCP-1, Second-eye cataract surgery, Diabetes, Aqueous humor, Inflammatory response

## Abstract

**Background:**

Substance P (SP) is a nociceptive tachykinin which regulates the immune inflammatory reactions including monocyte chemoattractant protein 1 (MCP-1) production. Sequential second-eye cataract surgery patients often suffer more pain than the first one partly because of the MCP-1 increase in aqueous humor (AH). This study aims to illustrate whether SP is involved in sympathetic inflammatory responses in the contralateral eye in patients with or without type 2 diabetes.

**Methods:**

This prospective randomized clinical study included 51 cataract patients (22 with diabetes and 29 without). Bilateral sequential cataract surgeries were conducted with 1-day or 1-week interval randomly. More than 100 μl of AH were obtained before surgery and stored for later analysis using magnetic Luminex assays to detect interleukin (IL)-1β, IL-1ra,IL-6, IL-8, IL-10, MCP-1, vascular endothelial growth factor, spinal macrophage inflammatory protein (MIP-1a), interferon-inducible protein 10 (IP-10), regulated on activation, normal T expressed and secreted (RANTES), as well as the enzyme-linked immunosorbent assay for SP.

**Results:**

Among the 4 groups, no significant differences were found in age, sex distribution, the R/L ration of the first surgery eye, or the lens nucleus hardness (*P* ≥ 0.802). Over 100 μl of AH samples were collected safely in all cases without intraoperative complications. SP and MCP-1 levels were both increased significantly in the second eye of the diabetic patients with 1-day and 1-week interval (*P* ≤ 0.040). The SP increase in the second eye of the diabetic patients were significantly higher than that of the patients without diabetes (*P* ≤ 0.030) both in the groups with 1-day and 1-week interval. Similarly, the MCP-1 increase was significantly higher in the diabetic patients in the group with 1-week interval (*P* = 0.042).

**Conclusions:**

Substance P and MCP-1 productions elevated in the AH of the contralateral eye after the first-eye cataract surgery in diabetic patients, indicating that SP and MCP-1 were involved in the sympathetic inflammatory responses. Diabetic patients are susceptible to noninfectious inflammation after cataract surgery, and perceive more pain in the second-eye phacoemulsification.

**Trial registration:**

Chinese Clinical Trial Registry, ChiCTR1900028374, retrospectively registered on 20th December, 2019.

## Background

Substance P (SP) is an undecapeptide member of the tachykinin family, acting as an neurotransmitter or neuromodulator in a wide range of physiological and pathophysiological processes in the central nervous system and peripheral tissues. It is mainly secreted by neurons, and a variety of nonneuronal cells including microglia, epithelial cells, endothelial cells, and immune cells [1]. SP plays a role in regulating the immune system, including inflammation [2, 3], apoptosis [4], and induction of the expression of the production of chemokines and pro-inflammatory cytokines [1, 5]. It is also involved in the pain nervous system as an important neurotransmitter mediating nociceptive transmission [6].

There is a clinical phenomenon that patients undergoing cataract surgery (phacoemulsification) in the second eye usually complain about, namely the increased pain compared to the first surgical eye days or weeks ago. It is reported that a sympathetic inflammatory effect could be induced since the level of monocyte chemoattractant protein 1 (MCP-1) becomes elevated significantly in the aqueous humor of the contralateral non-operated eye [7, 8]. MCP-1, also called chemokine (C-C motif) ligand 2 (CCL2), is a pain-related inflammatory cytokine that recruits several types of immune cells to the sites of inflammation in the condition of tissue injury or infection [9]. MCP-1 production could be regulated by SP concentrations in different cell types [10–12], indicating that SP can stimulate MCP-1 production which attracts inflammatory cells to specific sites. The previous studies focused on the changes of the pain-related inflammatory cytokines such as interleukin (IL)-1, IL-6,12 IL-8, spinal macrophage inflammatory protein (MIP), MCP-1, vascular endothelial growth factor (VEGF), and regulated on activation, normal T expressed and secreted (RANTES) before and after cataract surgery of the first eye, however the neuropeptide SP has not been identified. Thus in the current study we aimed to illustrate whether SP participated in the inflammatory reaction in the contralateral eye after the first-eye cataract surgery in patients with or without type 2 diabetes, which might illuminate the mechanism of sympathetic immune responses.

## Methods

### Subjects

This prospective consecutive randomized clinical study was conducted in the Eye Hospital of Wenzhou Medical University from July 2018 to December 2018, approved by the Institutional Review Board of the Affiliated Eye Hospital of Wenzhou Medical University (No. KYK[2018]24), and has been retrospectively registered in the Chinese Clinical Trial Registry (No. ChiCTR1900028374). The whole procedure adheres to CONSORT guidelines with a complete CONSORT checklist as an additional file. The written informed consent was obtained from all subjects before joining in the project.

Patients that were diagnosed with age-related cataract with or without type 2 diabetes and had the intention to fulfill the bilateral cataract surgeries in a short period were included, while the ones with a history of previous eye surgery, trauma, high intraocular pressure, glaucoma or shallow anterior chamber, high myopia, proliferative diabetic retinopathy or other retinal diseases were excluded. Similarly, eyes that failed to obtain > 100 μl aqueous humor (AH) or encountered intraopertative complications such as posterior capsule rupture and endothelial injury were excluded. Patients with or without type 2 diabetes were randomly assigned to 2 groups respectively: 1-day or 1-week surgical interval for the two eyes, so 4 groups were included in this study. The patient enrollment was conducted by two senior doctors, and the random sequence was determined by an assistant using the random number table.

All the cataract surgeries were fulfilled by the same surgeon (XH.G.). Aqueous humor samples (100–200 μl) were collected by inserting a 26-gauge needle into the anterior chamber before starting the surgery, and were immediately stored within a − 80 °C refrigerator. A standard surgical protocol included: a 2.0 mm clear corneal incision, standard phacoemulsification procedure, and a foldable IOL implantation. Postoperative medication is standardized using topical antibiotic-corticosteroid combination eye drops (Tobramycin 0.3%/ dexamethasone 0.1%; Alcon Laboratories, Fort Worth, Texas, USA) 4 times daily for 1-week. The eye drops were tapered and discontinued over 3 weeks. Follow-up visits were scheduled on 1-day, 1-week, and 1 and 3 months intervals postoperatively.

#### Magnetic Luminex assays

We used magnetic Luminex assays (Human Premixed Multi-Analyte Kit, R&D Systems, Inc., Minneapolis, MN, USA) to analyze 10 selected proteins simultaneously, including IL-1β, IL-1ra,IL-6, IL-8, IL-10, MCP-1, VEGF-A, MIP-1a, interferon-inducible protein 10 (IP-10) and RANTES. Analyte-specific antibodies were pre-coated onto color-coded magnetic microparticles. A total of 102 AH samples were drawn from both eyes of the 29 ARC patients and 22 diabetic patients, 50 μl AH was pipetted into the wells and the immobilized antibodies binded the specific proteins. After washing away the unbound substances, a biotinylated antibody cocktail was added to each well. Following a wash, streptavidin-phycoerythrin conjugate was added, then after a final wash, the microparticles were detected using the FLEXMAP 3D® Analyzer. Densitometric analysis of each spot was then performed using a xPONENT® software (Luminex® Corporation).

#### Elisa

The SP concentrations of the AH samples were tested by the Human SP ELISA kits (ab133029, Abcam, MA, USA). Specifically, 50 μl AH sample or standards were added to the wells, along with an alkaline phosphatase (AP) conjugated-SP antigen and a polyclonal rabbit antibody specific to SP. The plate was incubated at room temperature for 2 h, then after a wash, the pNpp substrate was added. After a incubation of 1 h at room temperature, stop solution was added and the plate was read immediately at 405 nm. The optical density was inversely proportional to the amount of SP captured in the plate.

#### Statistical analysis

SPSS software version 20.0 (SPSS Inc., Chicago, IL) was used. Comparisons of both eyes of the same patients utilized the paired t test to determine if the data is of normal distribution, or otherwise the Wilcoxon signed rank test was used with the Bonferroni correction. Comparisons of the differences (the second surgical eye minus the first one) between the ARC patients and diabetic patients were conducted using the independent t test to determine if the data is of normal distribution, or otherwise the Mann-Whitney U rank test was utilized. Two-tailed *P* < 0.05 were considered to indicate a statistically significant difference.

## Results

A total of 29 patients (15 women and 14 men; mean age, 68.52 ± 11.24 years) diagnosed with age-related cataract (ARC), and 22 patients (11 women and 11 men; mean age, 68.46 ± 9.94 years) with cataract and type 2 diabetes were recruited. They were randomly assigned to group 1: ARC patients with 1-day surgical intervals for the two eyes, group 2: ARC patients with 1-week surgical intervals, group 3: diabetic patients with 1-day surgical intervals, group 4: diabetic patients with 1-week surgical intervals. No significant differences were noted in the age, sex distribution, the R/L ration of the first surgery eye, or the lens nucleus hardness in each group (*P* ≥ 0.802, Table 1). No intraoperative complications, including the posterior capsular rupture, iris prolapse, iridemia or corneal endothelial injury occurred in the total 51 patients.

**Table 1:** Preoperative clinical characteristics of the subjects in each group

	Age-related Cataract		Diabetic Cataract		*P* value
	1 Day (*n* = 15) 1 Week (*n* = 14)		1 Day (n = 11) 1 Week (*n* = 11)		
Age (years)	69.67 ± 9.71	67.29 ± 12.75	69.09 ± 10.92	68.18 ± 9.39	0.943
DM duration (years) /		/	6.23 ± 3.86	5.82 ± 3.40	0.795
M:F	8:7	6:8	5:6	6:5	0.924
R:L	6:9	7:7	7:4	5:6	0.802
Nuclear	2.27 ± 0.88	2.43 ± 0.94	2.55 ± 0.82	2.45 ± 0.93	0.880

*DM* Diabetes mellitus, *M* Male, *F* Female, *R* Right eye, *L* Left eye; Note: the *P* value is the smallest value in the comparison between 2 groups

### Substance P level increased in the 2nd surgical eye in DM patients

**Fig. 1:**
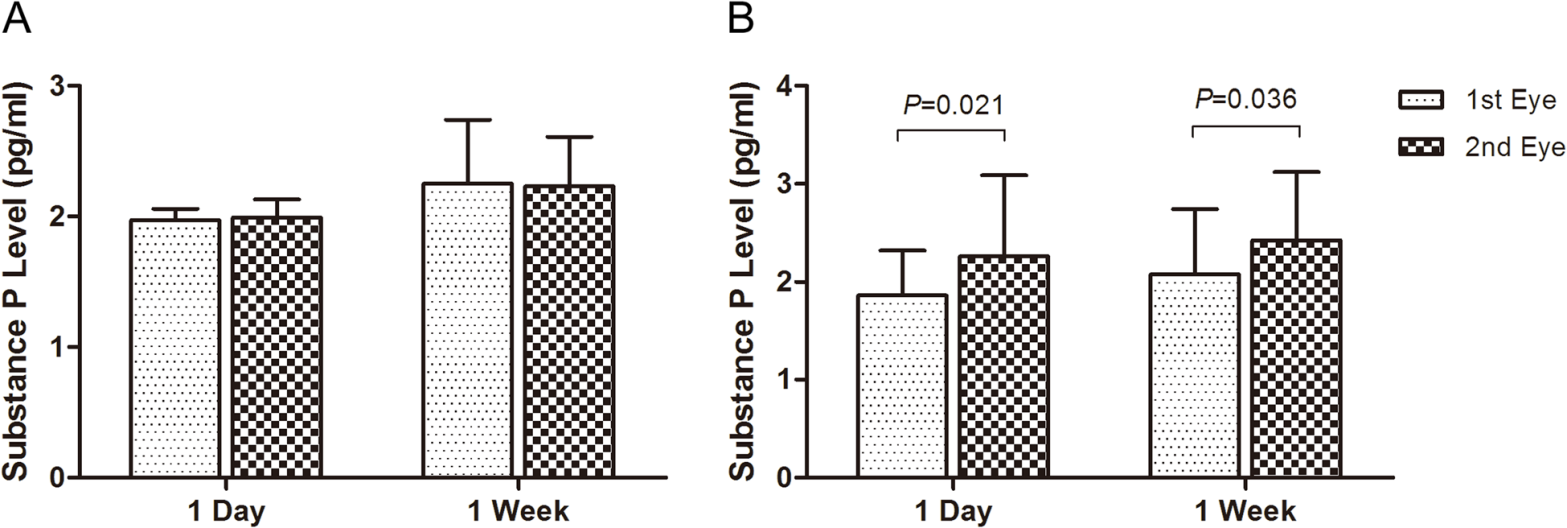
The increase of substance P level in the aqueous humor of the second surgical eye in the 4 groups. For the age-related cataract (ARC) patients with 1-day and 1-week surgical interval, SP level did not change after the first-eye surgery (**a**). While for the diabetic patients, it increased significantly in the second eye both with 1-day and 1-week surgical interval (*P* ≤ 0.036) (**b**)

For the ARC patients in group 1, the substance P level was 1.97 ± 0.09 pg/ml in the first surgical eye, and 1.99 ± 0.14 pg/ml in the second eye (*P* = 0.484) (Fig. 1, Table 2); in group 2, it was 2.25 ± 0.49 pg/ml and 2.23 ± 0.38 pg/ml in the first and second eye respectively (*P* = 0.747) (Fig. 1, Table 2). While for the diabetic patients in group 3, the SP level was 1.86 ± 0.46 pg/ml in the first eye, and 2.26 ± 0.83 pg/ml in the second one, which was significantly increased by 0.40 ± 0.48 pg/ml (t = 2.724, *P* = 0.021) (Fig. 1, Table 3); and in group 4, it was 2.08 ± 0.66 pg/ml and 2.42 ± 0.70 pg/ml in the first and second eye respectively with a significant increase of 0.34 ± 0.48 pg/ml (t = 2.356, *P* = 0.040) (Fig. 1, Table 3).

**Table 2:** The cytokine levels of the first and the second surgery eyes of age-related cataract in the aqueous humor

Age-related Cataract		SP pg/ml	MCP-1 ng/ml	IL-1β pg/ml	IL-1 ra pg/ml	MIP-1 pg/ml	RANTES pg/ml	IL-8 pg/ml	VEGF-A pg/ml
1st eye 2nd eye (1 D) 1st eye 2nd eye (1 W)	M ± SD M ± SD *P* M ± SD M ± SD *P*	1.97 ± 0.09 1.99 ± 0.14 0.484 2.25 ± 0.49 2.23 ± 0.38 0.747	0.43 ± 0.13 0.49 ± 0.13 0.184 0.47 ± 0.12 0.49 ± 0.12 0.645	2.09 ± 0.19 1.99 ± 0.18 0.223 2.03 ± 0.19 1.97 ± 0.15 0.414	65.7 ± 41.7 90.6 ± 140 0.464 40.6 ± 25.6 56.8 ± 49.4 0.075	84.0 ± 11.5 78.5 ± 16.7 0.309 79.1 ± 20.2 77.0 ± 14.8 0.788	8.07 ± 3.38 6.70 ± 2.83 0.095 4.43 ± 3.63 3.87 ± 3.21 0.548	8.07 ± 3.38 6.70 ± 2.83 0.095 4.43 ± 3.63 3.87 ± 3.21 0.548	58.46 ± 19.83 61.05 ± 20.45 0.705 64.54 ± 23.29 71.62 ± 23.51 0.220

**Table 3:** The cytokine levels of the first and the second surgery eyes of diabetic cataract in the aqueous humor

Diabetic Cataract		SP pg/ml	MCP-1 ng/ml	IL-1β pg/ml	IL-1 ra pg/ml	MIP-1 pg/ml	RANTES pg/ml	IL-8 pg/ml	VEGF-A pg/ml
1st Eye 2nd Eye (1 Day) 1st Eye 2nd Eye (1 Week)	M ± SD M ± SD *P* M ± SD M ± SD *P*	1.86 ± 0.46 2.26 ± 0.83 0.021 2.08 ± 0.66 2.42 ± 0.70 0.040	0.41 ± 0.10 0.50 ± 0.11 0.025 0.52 ± 0.15 0.63 ± 0.22 0.006	2.74 ± 0.18 3.09 ± 0.91 0.260 2.74 ± 0.25 2.92 ± 0.29 0.160	83.9 ± 40.6 109 ± 92.2 0.444 89.0 ± 67.9 62.5 ± 32.9 0.245	68.2 ± 24.4 65.8 ± 17.1 0.804 70.0 ± 27.5 63.4 ± 20.2 0.558	2.50 ± 1.89 2.70 ± 2.29 0.817 2.05 ± 0.94 2.51 ± 0.55 0.102	2.50 ± 1.89 2.70 ± 2.29 0.817 2.05 ± 0.94 2.51 ± 0.55 0.102	71.60 ± 40.75 65.69 ± 25.35 0.593 75.95 ± 20.18 66.73 ± 21.16 0.194

### MCP-1 level increased in the 2nd surgical eye in DM patients

**Fig. 2:**
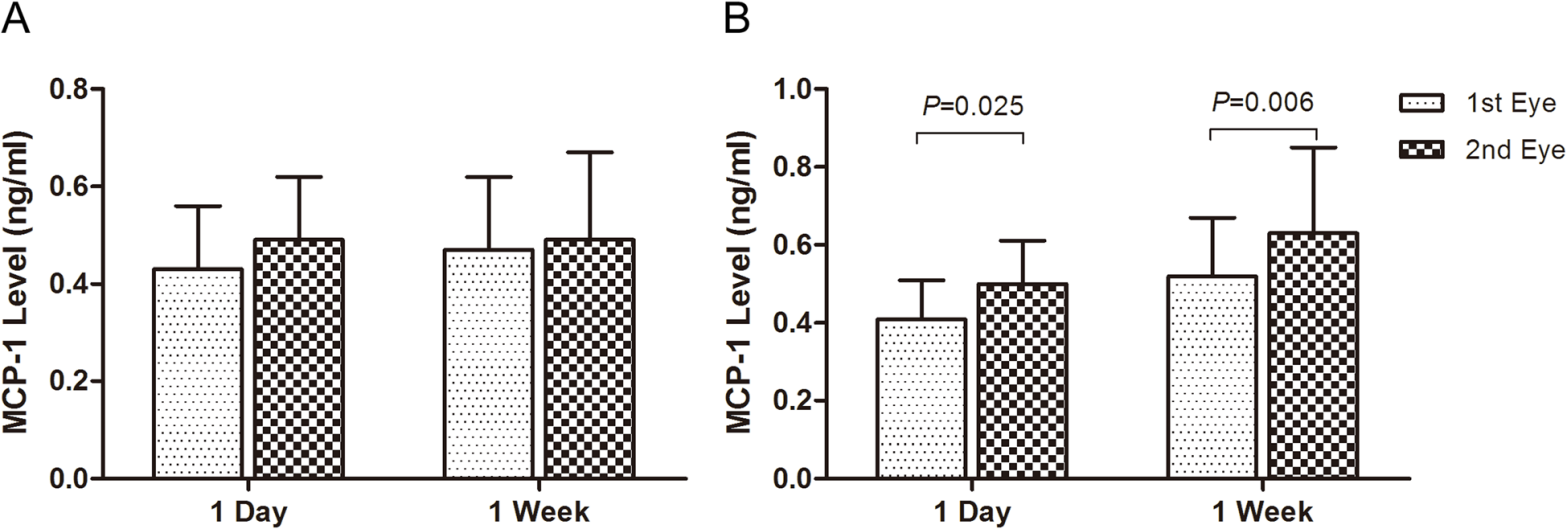
The increase of MCP-1 level in the aqueous humor of the second surgical eye in the 4 groups. MCP-1 production did not change significantly in the two eyes for the ARC patients with 1-day and 1-week interval (**a**). It increased significantly in that of the diabetic patients with 1-day and 1-week interval (*P* ≤ 0.025) (**b**)

There were no significant differences of the MCP-1 level between the first and the second surgical eyes for the ARC patients both in group 1 and group 2 (*P* ≥ 0.184) (Fig. 2, Table 2). However for the diabetic patients in group 3, the MCP-1 level was 0.41 ± 0.10 ng/ml in the first eye and 0.50 ± 0.11 ng/ml in the second eye with a significant increase of 0.09 ± 0.12 ng/ml (t = 2.642, *P* = 0.025) (Fig. 2, Table 3); and in group 4, it also significantly increased from 0.52 ± 0.15 ng/ml in the first eye, to 0.63 ± 0.22 ng/ml in the second eye(t = 3.496, *P* = 0.006) (Fig. 2, Table 3).

### The relative differences between the ARC & DM patients

**Fig. 3:**
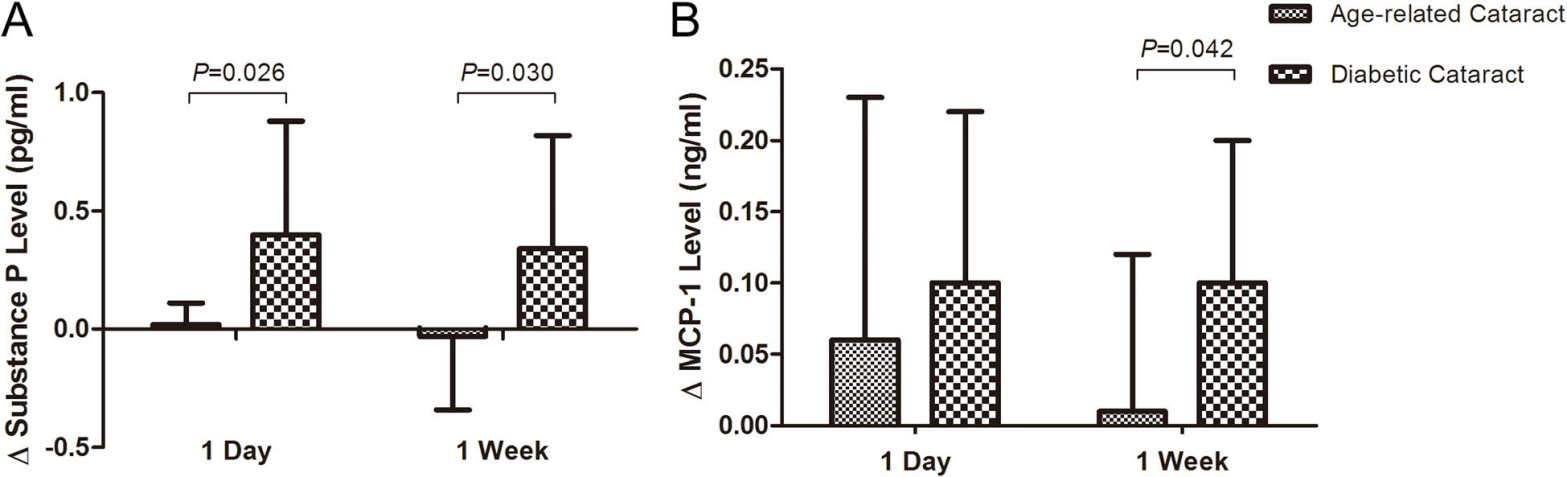
The binocular differences between the ARC and diabetic patients. The binocular differences of SP of the diabetic patients were significantly higher than those of the ARC patients (*P* ≤ 0.030) both with 1-day and 1-week surgical interval (**a**). That of MCP-1 was significantly higher in the diabetic patients in the group with 1-week interval (*P* = 0.042) (**b**)

The SP and MCP-1 levels increased significantly in the second surgical eye of the diabetic patients compared with those of the ARC patients. The difference of SP was 0.38 ± 0.15 pg/ml (t = 2.593, *P* = 0.026) for the ARC and diabetic groups with 1-day interval, and 0.37 ± 0.16 pg/ml (t = 2. 312, *P* = 0.030) for those with 1-week interval (Fig. 3a). Although the difference of MCP-1 was not significant for the two groups with the 1-day interval (0.03 ± 0.06 ng/ml, *P* = 0.583), it was significantly different for those with 1-week interval (0.09 ± 0.104 ng/ml, t = 2. 152, *P* = 0.042) (Fig. 3b). For the diabetic patients, there was no difference in the increases of the SP levels in the second surgical eye between the 1-day and 1-week groups (*P* = 0.777).

## Discussion

Based on the high quality, safety and efficiency of modern cataract surgery, more and more patients choose to complete the bilateral sequential cataract surgery in a short period. It has been reported that the second eye may suffer more pain during surgery under topical anesthesia relative to the first surgical eye [13–16], since the inflammatory responses could be activated with increased levels of MCP-1 and transforming growth factor beta 2 (TGF-β2) in the AH of the contralateral eye [7, 8, 17]. In the current study, we compared the expressions of 11 inflammation-related proteins in the AH of the cataract patients with and without type 2 diabetes before and after the first-eye surgery, and revealed that the first-eye surgery in the diabetic patients stimulated significant increases in the levels of MCP-1 and the neuropeptide SP in the contralateral eye. While the differences between the two eyes in the ARC patients seems to be insignificant, this indicated that more sympathetic immune responses could be induced in the diabetic patients after the initial ctaract surgery.

Although predominantly released from sensory nerve fibers, SP is also produced by epithelial cells, endothelial cells, and immune cells, in which it interacts with neurokinin receptors (NKR) to regulate their biological functions in an autocrine or paracrine manner [2]. SP is found to play a potentially protective role in ocular inflammation, wound healing and tissue homeostasis, especially in diabetic organisms [2, 18]. SP facilitates regeneration of impaired corneal nerve fibers, promotes epithelial wound healing in diabetic mice, and also speeds up neuronal recovery in vitro under hyperglycemic conditions [18]. It is reported that insults like retinal laser burns or unilateral ultraviolet radiation in one eye subsequently caused NKR-1 upregulation in both eye tissues in animal models [19, 20], indicating that SP-NKR interaction may have a bilateral crosstalk when one eye was injured. Our results showed that SP expression increased significantly in the contralateral eye 1-day or 1-week after the first-eye cataract surgery in the diabetic patients but not in normal patients, demonstrating that SP-related immune responses could be activated more easily in diabetic patients after one-eye cataract surgery. These responses may play a protective role for diabetic patients since temperate increase in SP production could enhance wound healing and anti-infection ability [18, 21, 22]. Besides, SP can protect cells from oxidantive damage [23] while hyperglycemia is a risk factor for oxidative stress [24].

MCP-1 actually has both roles of proinflammatory and anti-inflammatory effects through the interaction with its two types of receptors in different immune cells [9]. MCP-1 has been found to be one of the factors responsible for the increased pain perception during the sequential cataract surgery [7, 8], however there are also other reports that found no significant difference in pain perception between the first and second eye procedures [14, 25]. It is recognized that hyperglycemia prominently elevates MCP-1 expression in vitro and in vivo [26, 27]. Our current study demonstrated that MCP-1 level had no significant increase in the sequential surgical eye of the ARC patients, but that of the diabetic patients was significantly different, which coincides with the changes of SP. SP and MCP-1 might synergistically play a protective role by reducing hyperglycemia-induced oxidative injury. The current results may be due to the limited sample size, however it is suggestive that the diabetic patients are more susceptible to inflammatory reactions after first-eye cataract surgery, which might be associated with the sympathetic SP increase and the relevant MCP-1 upregulation in the AH.

Based on these results, it raises the question of how surgeons schedule their cataract patients with type 2 diabetes. Within this study, there was no difference between SP levels at the 1-day and 1-week intervals in diabetic patients. If elevated SP and MCP-1 have the potential to increase recovery and healing in the subsequent eye following the initial cataract extraction in diabetic patients, then there is the potential to develop surgical schedules for optimal intervals to utilize this inflammatory response for its suggested healing value. Further studies investigating longer intervals between the surgeries of both eyes are needed to determine the duration of SP and MCP-1 elevation within the aqueous humor, as well as the surgical outcomes of diabetic patients with higher SP versus lower SP.

## Conclusions

The current study reveals that Substance P and MCP-1 expressions were elevated synergistically in the AH of the contralateral eye after the first-eye cataract surgery in the diabetic patients, indicating that SP and MCP-1 related immune responses were involved in the sympathetic inflammation subsequent to the first-eye cataract surgery in the diabetic patients. This may explain why diabetic patients are more inclined to encounter noninfectious inflammatory response after cataract surgery, and suffer more pain in the second-eye phacoemulsification. It is also informative for cataract surgeons to schedule the bilateral cataract surgeries in type 2 diabetic patients.

## Data Availability

The datasets used and analysed during the current study are available from the corresponding author on reasonable request.

## Abbreviations

SP: Substance P
MCP-1: Monocyte chemoattractant protein 1
AH: Aqueous humor
IL: Interleukin
MIP: Macrophage inflammatory protein
VEGF: Vascular endothelial growth factor
IP-10: Interferon-inducible protein 10
RANTES: Regulated on activation, normal T expressed and secreted
CCL2: Chemokine (C-C motif) ligand 2
AP: Alkaline phosphatase
ARC: Age-related cataract
DM: Diabetes mellitus
TGF-β2: Transforming growth factor beta 2
NKR: Neurokinin receptors
M: Male
F: Female
R: Right eye
L: Left eye
